# Clinical characteristics of pulmonary embolism at extremely high altitude: a single-center retrospective study

**DOI:** 10.3389/fpubh.2025.1453700

**Published:** 2025-03-27

**Authors:** Jialin Wu, Jianli Zhang, Rui Wang, Xiaobo Han, Wan Wang, Qingqing Chen, Yali Gao, Munire Wusifujiang, Peng Jiang

**Affiliations:** ^1^Department of Respiratory Disease, General Hospital of Xinjiang Military Command, Ürümqi, China; ^2^Reproduction Medicine Center of General Hospital of Xinjiang Military Command, Ürümqi, China; ^3^Department of Emergency, General Hospital of Xinjiang Military Command, Ürümqi, China; ^4^Graduate School of Xinjiang Medical University, Ürümqi, China

**Keywords:** extremely high altitude, venous thromboembolism, pulmonary embolism, uric acid, hypoxia, risk factor

## Abstract

**Background:**

Exposure to high altitude (HA) has been shown to significantly increase the risk of venous thromboembolism (VTE). However, the clinical characteristics of VTE at extremely high altitudes remain poorly understood. In this single-center retrospective study, we aimed to compare the clinical characteristics and prognoses of pulmonary embolism (PE) patients at extremely high altitudes with those at low altitudes (LA).

**Methods:**

This retrospective analysis focused on PE patients treated at the General Hospital of Xinjiang Military Command between November 1, 2019, and November 1, 2022. The high-altitude group (HA-Group) consisted of patients who sought medical treatment after they had fallen ill into the plateau area, and the low-altitude group (LA-Group) consisted of local residents.

**Results:**

We identified a total of 17 PE patients in the HA-Group and 62 patients in the LA-Group. The average altitude in the HA-Group was 5,041 ± 85.34 m, and 802.1 ± 11.10 m in the LA-Group (*p* < 0.0001). Hematological indicators, including red blood cells, lymphocytes, platelet counts, hemoglobin, PT, APTT, the INR and uric acid, were significantly higher in the HA-Group than in the LA-Group. Kaplan–Meier curve analysis demonstrated that the time to complete resolution of pulmonary thrombosis was significantly shorter in the HA-Group than in the LA-Group (log-rank *p* = 0.033).

**Conclusion:**

This retrospective study revealed the clinical characteristics of PE patients at extremely high altitudes. High-altitude exposure may increase the susceptibility of young people to PE, and abnormal serum uric acid metabolism may be a potential risk factor for PE in high altitude areas.

## Background

Venous thromboembolism (VTE), which primarily comprises pulmonary embolism (PE) and deep venous thrombosis (DVT) ranks among the top five most common vascular diseases in many countries (1). In China, the age- and sex-adjusted hospitalization rate for VTE is 17.5 per 100,000 people, reflecting a significant increase over the past decade (2). The incidence of VTE in other parts of Asia shows a similar pattern. In the USA and Europe, VTE incidence rates are believed to be higher than those in Asia, with an estimated range of 1–2 cases per 1,000 person-years (3). Naturally, the incidence rates of VTE vary widely across different regions due to variations in age, sex, race, and medical conditions.

PE and DVT are different stages of the same pathological process, and a majority of PE patients experience DVT. Even if DVT is not detected in PE patients, it is possible that the entire thrombus has detached or that it is not detectable by the instrument (4). Numerous mechanisms or pathological processes have been shown to directly or indirectly contribute to venous thrombosis. Depending on the underlying pathological mechanism, risk factors for the development of VTE are often classified as acquired (such as cancer, major surgery, trauma, advanced age, reduced mobility, pregnancy, and postpartum period, etc.) or hereditary (including plasminogen deficiency, antithrombin deficiency, protein S/C deficiency, and prothrombin gene mutation, etc.) (4). In epidemiological studies, events that contribute to VTE are categorized as provoked or unprovoked. The former refers to events occurring within 3 months of specific triggering factors (such as infection, surgery, trauma, fracture, injuries, etc.), while the latter occurs in the absence of these conditions (5).

High altitude areas, as unique geographical environments, have attracted significant amounts of attention because of their role in thrombotic diseases. The incidence of VTE caused by hypoxia, which is the primary trigger, is significantly greater in high altitude regions than in lower altitudes (6–10). The high altitude environment can contribute to disturbances in coagulation and increase the risk of thrombosis, either as an acquired or provoked factor within the body’s internal environment. Detecting and diagnosing venous thrombotic diseases in high altitude areas presents certain challenges due to the nonspecific symptoms these diseases present and their similarity to altitude-related ailments (11).

To address this issue, the present study utilized data from a single center to assess the clinical characteristics, hematological status, prognosis, and other pertinent factors of PE patients residing in both extremely high-altitude regions and plains. The focus of the study was to observe trends in characteristic indicators throughout the progression of the disease in PE patients from both regions. The results obtained from this research endeavor are expected to increase awareness of the occurrence of venous thrombotic diseases in high altitude areas.

## Methods

### Patients

This retrospective cohort study included patients from the General Hospital of Xinjiang Military Command (Urumqi, China, 800 m) between November 1, 2019, and November 1, 2022. The inclusion criteria were as follows: (1) patients diagnosed according to the International Classification of Diseases (ICD-9/10) standards; (2) diagnosis of pulmonary embolism (PE) confirmed by computed tomography pulmonary angiography (CTPA) showing pulmonary artery filling defects; (3) age older than 18 years; (4) the HA-Group patients had a clear history of high-altitude exposure and could provide relevant information about their history of high-altitude exposure (such as duration of exposure and history of acute or chronic high-altitude illness). Exclusion criteria: (1) incomplete CTPA examination during hospitalization. (2) Age younger than 18 years. (3) Patients were already diagnosed with PE before November 1, 2019. (4) Information on the altitude at the time of onset could not be provided. All the data were collected jointly by two researchers, and if there was any disagreement, a third researcher was consulted.

### Grouping and laboratory examination

Patients who developed PE in high altitude areas were classified as the HA-Group; otherwise, they were classified as the LA-Group. Given that the average age of the HA-Group was significantly younger than that of the LA-Group, patients aged younger than 60 years in the LA-Group were defined as the LA-Y-Group to reduce the heterogeneity caused by age. To avoid errors caused by the use of therapeutic drugs, the preferred approach for collecting hematological examination results is to use samples collected before treatment or blood samples obtained during the initial stage of the disease course. As most patients in the HA-Group develop the disease in high-altitude areas and are referred to our hospital from other hospitals, some of the test results were obtained from other medical institutions.

### Follow-up and prognosis

The end point of follow-up observation for pulmonary thromboembolism was set at 1 year after diagnosis in this study. The diagnosis of thrombus resorption relies on CTPA. If there was no follow-up record for the patient at our hospital, a telephone follow-up was conducted. Patients for whom follow-up records could not be obtained even after further telephone follow-up were considered lost to follow-up.

### Statistical analysis

The data analysis was performed using Prism 6.0c software (GraphPad). Continuous data are presented as the mean ± standard deviation (SD) and were compared using a t test for normally distributed continuous variables. Nonparametric analyses, including the Mann–Whitney *U* test and Kruskal–Wallis test, were used when appropriate. Categorical variables are presented as *n* (%), and the normality of the data was assessed using the Shapiro–Wilk test. Comparisons of categorical data were conducted using the chi-square test or Fisher’s exact test. Survival curves for thrombolysis were generated using the Kaplan–Meier method, and differences were compared using the log-rank test. *p* < 0.05 were considered significant (*p < 0.05; **p < 0.01; ***p < 0.001; ****p < 0.0001); *p* > 0.05: no significant (ns).

## Results

### Patient demographics

**Figure 1:**
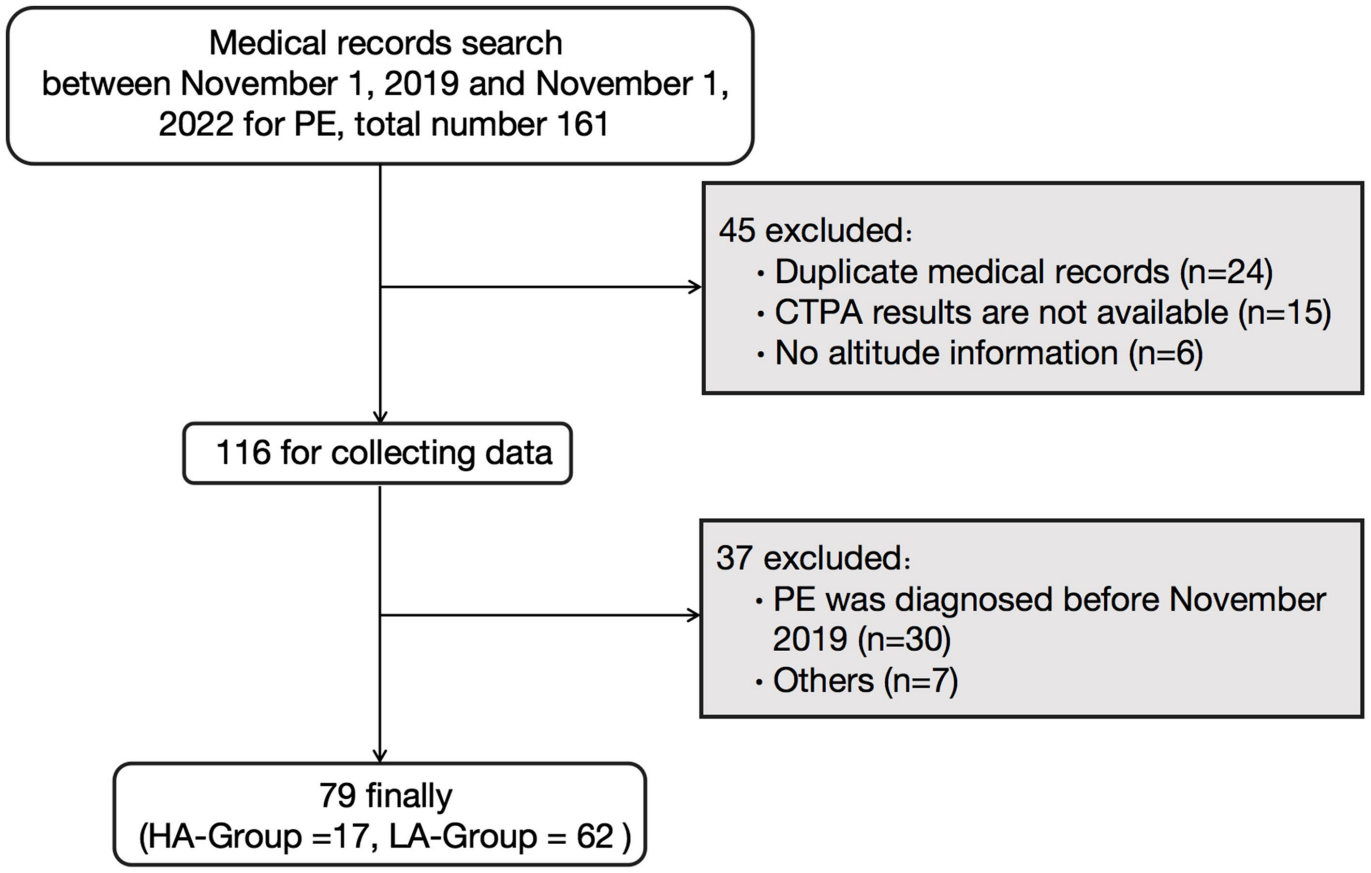
Flowchart of the study. PE, pulmonary embolism; CTPA, computer tomography pulmonary angiography; HA-Group, high altitude group; LA-Group, low altitude group.

Initially, 161 patients diagnosed with PE were identified. After applying the exclusion criteria, 79 patients were included in the study (Figure 1). The HA-Group comprised 17 individuals with a mean age of 24.35 ± 0.88 years, all of whom were males who had migrated from the plains to high-altitude regions (Qinghai-Xizang plateau). Due to the harsh environmental conditions, large-scale migration to extremely high-altitude regions (>5,000 m) is rare except for specific situations. The patients in the HA group were all from a population that migrated from low-altitude areas to extremely high-altitude regions for training missions, and the majority of this group were male. The average duration from arrival at high altitude to onset of symptoms was 125.1 ± 28.26 days, and the average altitude at the time of onset was 5,041 ± 85.34 m. The LA-Group consisted of 62 individuals, including 40 males, with an average age of 60.39 ± 2.35 years and an average altitude of 802.1 ± 11.10 m (Table 1).

**Table 1:** Basic characteristics of patients.

Variables	HA-Group (*n* = 17)	LA-Group (*n* = 62)	*p*-value	*χ^2^*
Male (%)	17 (100%)	40 (64.5%)		
Age	24.35 ± 0.88	60.39 ± 2.35	<0.0001	
Male (years, mean ± SD)	24.35 ± 0.88	55.73 ± 3.05	<0.0001	
Female (years, mean ± SD)		68.86 ± 2.88		
Altitude (m, mean ± SD)	5,041 ± 85.34	802.1 ± 11.10	<0.0001	
Time at HA (days, mean ± SD)	125.1 ± 28.26			
Ethnicity				
Han [*n* (%)]	14 (82.4%)	49 (79%)	0.091	0.763
Smoking [*n* (%)]	9 (52.9%)	25 (40.3%)	0.418	0.655
Alcohol consumption [*n* (%)]	1 (5.9%)	14 (22.6%)	0.228	1.455
Long-term bedridden [*n* (%)]	0	9 (14.5%)		
Long-term administration of glucocorticoid [*n* (%)]	0	3 (4.8%)		
Long-term administration of anticoagulant [*n* (%)]*	1 (5.9%)	11 (17.7%)	0.409	0.681
Surgical history within 2 years [*n* (%)]	0	9 (14.2%)		
The average time from onset of symptoms to diagnosis (days, mean ± SD)	19.59 ± 4.68	7.48 ± 0.9	<0.0001	
Underlying diseases [*n* (%)]				
Hypertension	0	26 (41.9%)		
Coronary heart disease	0	12 (19.4%)		
Lower limb varicosity	0	9 (14.51%)		
Diabetes	0	8 (12.9%)		
Chronic bronchiti	0	7 (11.3%)		
Cerebral infarction	0	6 (9.7%)		
Lower limb venous thrombosis	1 (5.9%)	6 (9.7%)	0.995	0
Malignant tumor	0	5 (8.1%)		
Chronic obstructive pulmonary disease	0	4 (6.5%)		
Chronic heart failure	0	4 (6.5%)		
Atrial fibrillation	0	5 (8.1%)		

HA-Group, high altitude group; LA-Group, low altitude group; HA, high altitude. *The use of anticoagulants before the onset of the disease is for the treatment of lower extremity venous thrombosis and the prevention of atrial fibrillation.

Due to the younger age of the HA-Group, there were significantly fewer underlying diseases, with only one patient having a history of lower extremity venous thrombosis. In contrast, the LA-Group had a significantly greater proportion of patients with underlying disease, such as hypertension, coronary heart disease. In terms of the average duration from symptom onset to definitive diagnosis, the HA-Group was significantly longer than the LA-Group.

### Clinical characteristics of patients

In terms of clinical symptoms, there was a statistically significant difference in the proportion of patients with only hemoptysis between the two groups, while the proportions of patients with other symptoms were not significantly different. However, in terms of the frequency of symptom occurrence, common symptoms in the HA-Group included chest pain, panting, pain and swelling in the lower limb and hemoptysis. In the LA-Group, common symptoms included chest pain, panting, and cough. Moreover, there was no statistically significant difference between the two groups in terms of the incidence of respiratory failure, pulmonary embolism, or mortality due to PE. Notably, in the HA-Group, seven individuals had concomitant high altitude pulmonary edema (HAPE) (Table 2).

**Table 2:** Clinical features of patients.

Variables	HA-Group Total number = 17 [*n* (%)]	LA-Group Total number = 62 [*n* (%)]	*p*-value	*χ^2^*
Death	1 (5.9%)	6 (9.7%)	0.995	0
Death caused by PE directly	1 (5.9%)	1 (1.6%)	0.903	0.015
Respiratory failure	2 (11.8%)	17 (27.4%)	0.256	1.290
Pulmonary infarction	5 (29.4%)	10 (16.1%)	0.375	0.789
Comorbidities				
DVT	10 (58.8%)	33 (53.2%)	0.955	0.003
CVST	2 (11.8%)	1 (1.6%)	0.221	1.498
Renal venous thrombosis	1 (5.9%)	0		
HAPE	7 (41.2%)	0		
Symptoms				
Chest pain	7 (41.2%)	20 (32.2%)	0.492	0.472
Panting	6 (35.3%)	26 (41.9%)	0.621	0.244
Cough	5 (29.4%)	18 (29%)	1	0.026
Expectoration	4 (23.5%)	13 (21%)	1	0
Dyspnea	2 (11.8%)	6 (9.7%)	1	0
Pain and swelling in the lower limb	6 (35.3%)	6 (9.7%)	0.026	4.953
Nausea	3 (17.6%)	6 (9.7%)	0.267	0.236
Vomiting	3 (17.6%)	3 (4.8%)	0.212	1.561
Hemoptysis	6 (35.3%)	4 (6.5%)	0.006	7.600
Intravenous thrombolytic therapy	1 (5.9%)	2 (3.2%)	0.609	0.262
Treatment options outside the hospital				
Warfarin (oral)	8/16 (50%)	14/56 (25%)		
Rivaroxaban (oral)	7/16 (43.8%)	40/56 (71.4%)		
Dabigatran (oral)	0	1/56 (1.8%)		
No anticoagulant therapy	0	1/56 (1.8%)		

HA-Group, high altitude group; LA-Group, low altitude group; PE, pulmonary embolism; HAPE, High altitude pulmonary edema; DVT, deep venous thrombosis; CVST, cerebral venous sinus thrombosis.

**Figure 2:**
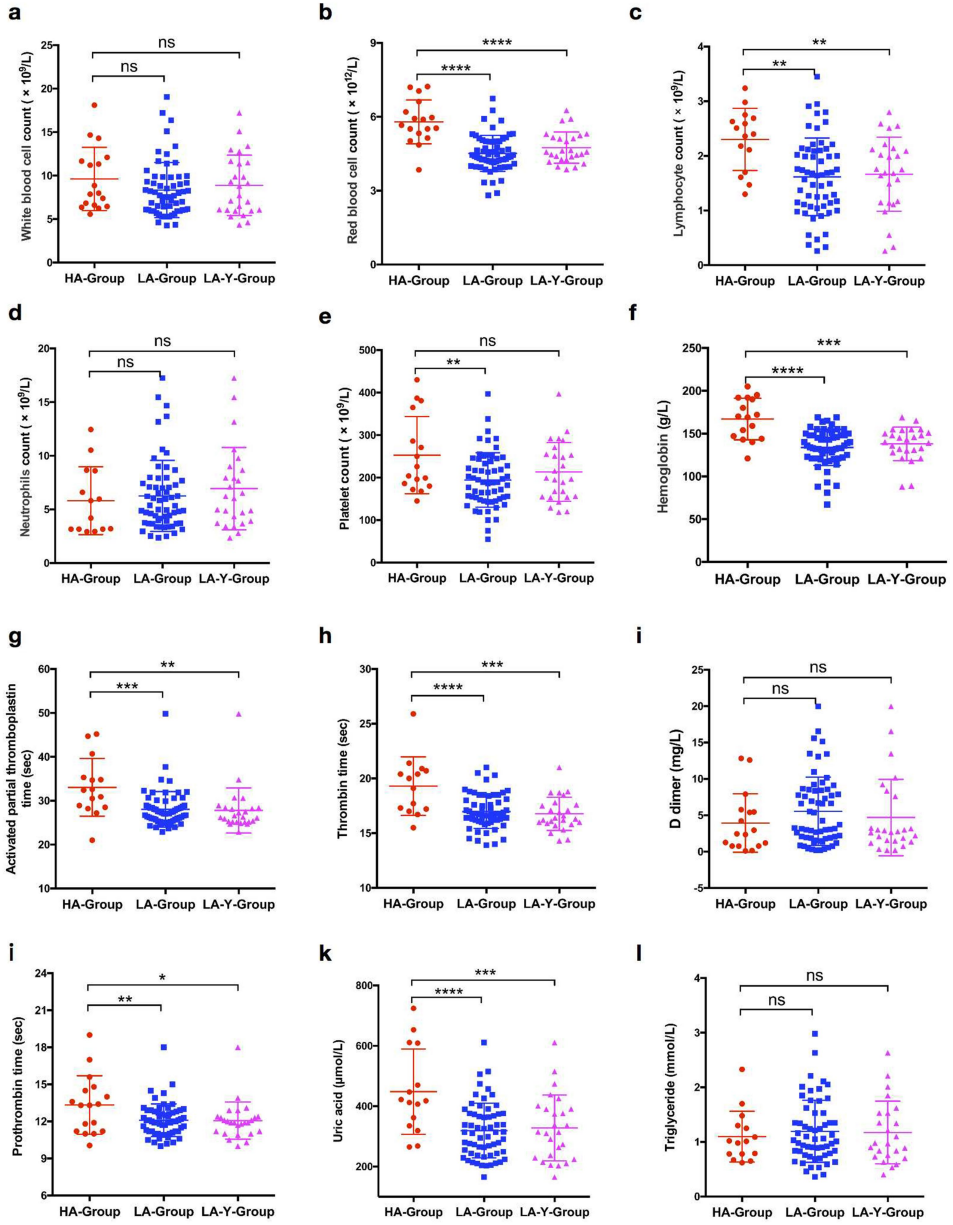
Comparison of biochemical indexes expression levels in patients between HA-Group, LA-Group, and LA-Y-Group. **(a)** White blood cell counts in HA-Group (*n* = 17), LA-Group (*n* = 61), and LA-Y-Group (*n* = 26). **(b)** Red blood cell counts in HA-Group (*n* = 17) and LA-Group (*n* = 61). **(c)** Lymphocyte counts in HA-Group (*n* = 15), LA-Group (*n* = 60), and LA-Y-Group (*n* = 26). **(d)** Neutrophils counts in HA-Group (*n* = 14), LA-Group (*n* = 61), and LA-Y-Group (*n* = 26). **(e)** Platelet counts in HA-Group (*n* = 16), LA-Group (*n* = 61), and LA-Y-Group (*n* = 26). **(f)** Hemoglobin expression level in HA-Group (*n* = 16), LA-Group (*n* = 62), and LA-Y-Group (*n* = 26). **(g)** Activated partial thromboplastin time in HA-Group (*n* = 15), LA-Group (*n* = 62), and LA-Y-Group (*n* = 26). **(h)** Thrombin time in HA-Group (*n* = 15), LA-Group (*n* = 62), and LA-Y-Group (*n* = 26). **(i)** D dimer in HA-Group (*n* = 17), LA-Group (*n* = 62), and LA-Y-Group (*n* = 26). **(j)** Prothrombin time in HA-Group (*n* = 17), LA-Group (*n* = 62), and LA-Y-Group (*n* = 26). **(k)** Uric acid expression level in HA-Group (*n* = 15), LA-Group (*n* = 61), and LA-Y-Group (*n* = 26). **(l)** Triglyceride expression level in HA-Group (*n* = 15), LA-Group (*n* = 57), and LA-Y-Group (*n* = 24).

According to the initial hematological examination results, there were significant differences in several parameters between the two groups. Hematological indicators such as red blood cell counts, lymphocyte counts, platelet counts, hemoglobin levels, clotting-related indices such as PT, APTT, the INR, and uric acid levels were significantly higher in the HA-Group than in the LA-Group. However, there were no significant differences in the triglyceride levels, total white blood cell count, and D-dimer level. A similar trend was also observed in the HA-Group and LA-Y-Group (Table 3; scatter diagram is shown in Figure 2).

**Table 3:** Biochemical index profiles of patients.

Variables	HA-Group Total number = 17 [mean ± SD (*n*)]	LA-Group Total number = 62 [mean ± SD (*n*)]	*p*-value
WBC count (×10^9^/L)	9.60 ± 0.88 (*n* = 17)	8.33 ± 0.41 (*n* = 61)	0.157
Neutrophil count (×10^9^/L)	5.81 ± 0.84 (*n* = 14)	6.26 ± 0.42 (*n* = 61)	0.65
Lymphocyte count (×10^9^/L)	2.30 ± 0.15 (*n* = 15)	1.67 ± 0.10 (*n* = 60)	<0.01
Monocyte count (×10^9^/L)	0.58 ± 0.06 (*n* = 14)	0.55 ± 0.03 (*n* = 61)	0.941
RBC count (×10^12^/L)	5.80 ± 0.22 (*n* = 17)	4.51 ± 0.09 (*n* = 61)	<0.0001
Hemoglobin (g/L)	167.1 ± 6.05 (*n* = 16)	133.9 ± 2.68 (*n* = 62)	<0.0001
Platelet count (10^9^/L)	252.9 ± 22.75 (*n* = 16)	194.5 ± 8.20 (*n* = 61)	<0.01
Fibrinogen (g/L)	3.94 ± 0.51 (*n* = 16)	3.74 ± 0.16 (*n* = 62)	0.622
PT (sec)	13.33 ± 0.57 (*n* = 17)	12.09 ± 0.17 (*n* = 62)	<0.01
TT (sec)	18.28 ± 1.22 (*n* = 15)	16.97 ± 0.20 (*n* = 62)	0.067
APTT (sec)	33.04 ± 1.698 (*n* = 15)	28.04 ± 0.5131 (*n* = 62)	<0.001
INR	1.14 ± 0.052 (*n* = 15)	1.01 ± 0.01 (*n* = 62)	<0.0001
D dimer (mg/L)	3.94 ± 0.97 (*n* = 17)	5.55 ± 0.60 (*n* = 62)	0.201
Uric acid (μmol/L)	448.1 ± 36.41 (*n* = 15)	319.2 ± 11.61 (*n* = 61)	<0.0001
Triglyceride (mmol/L)	1.10 ± 0.12 (*n* = 15)	1.19 ± 0.08 (*n* = 57)	0.544

HA-Group, high altitude group; LA-Group, low altitude group; WBC, white blood cell; RBC, red blood cell; PT, prothrombin time; TT, thrombin time; APTT, activated partial thromboplastin time; INR, international normalized ratio.

### Prognostic

**Figure 3:**
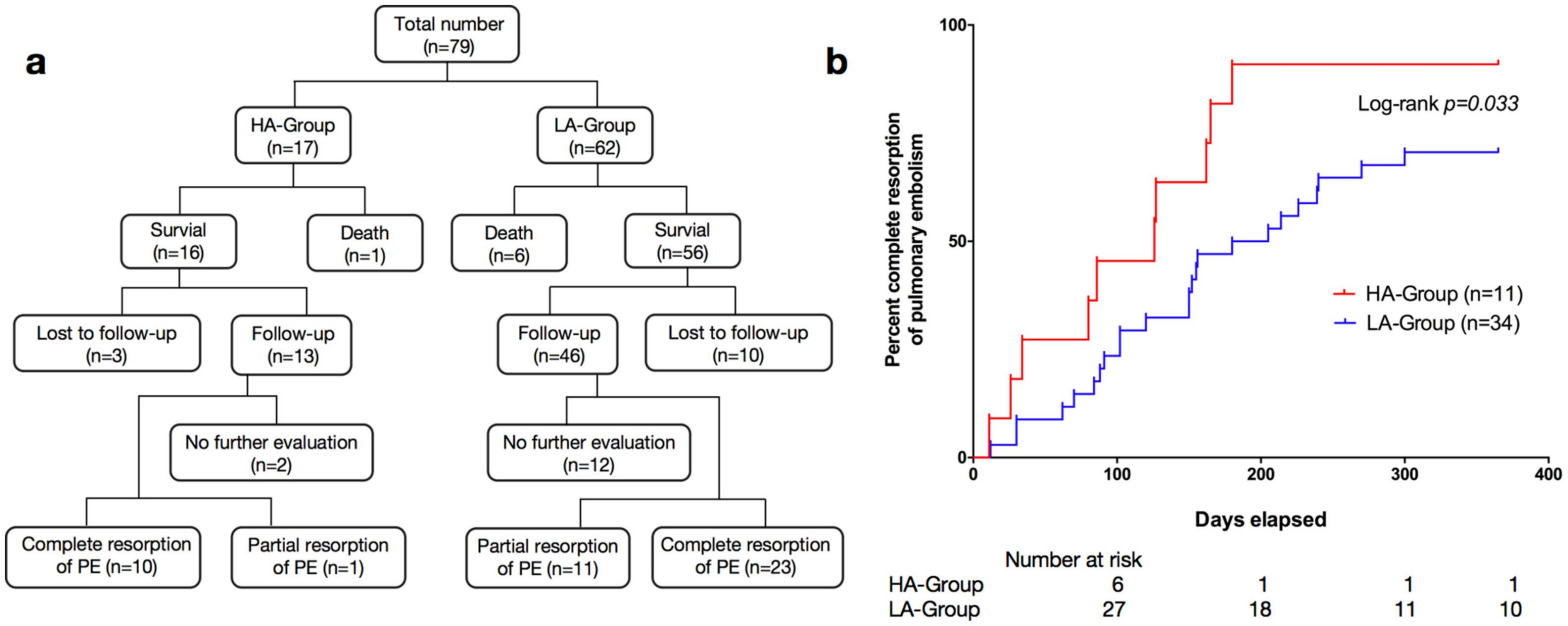
**(a)** Research flow diagram. The flow diagrams illustrate the trial profiles and research design. **(b)** Kaplan–Meier survival curves of pulmonary embolism patients: HA-Group (*n* = 11) vs. LA-Group (*n* = 34).

Seventeen patients in the HA-Group and 62 patients in the LA-Group were included in the follow-up process, with 3 and 10 individuals lost to follow-up in each group, respectively (Figure 3a). One year after the first definitive diagnosis of PE, CTPA indicated complete resorption of pulmonary thromboemboli in 10 patients in the HA-Group and 23 patients in LA-Group. Kaplan–Meier curve analysis demonstrated that the time to complete resolution of pulmonary embolism was significantly shorter in the HA-Group (log-rank *p* = 0.033; Figure 3b).

## Discussion

The epidemiology and pathogenesis of VTE in high-altitude regions have been prominent topics in high-altitude medicine in recent years. Exposure to high altitudes is increasingly recognized as a significant risk factor for VTE, regardless of whether individuals are in normal or diseased physiological states (12–15). This study compared the clinical data of PE patients at different altitudes in the years from 2019 to 2022. The main highlight of the study is that it focused on PE patients in extremely high altitude areas, and the participants were not native residents but migrants from plain areas. Additionally, the altitude of this study was above 5,000 m, emphasizing the uniqueness of the clinical data. This study also identified the particular features of the epidemiology of PE in extremely high-altitude areas, providing new insights into the occurrence and development of such diseases.

**Figure 4:**
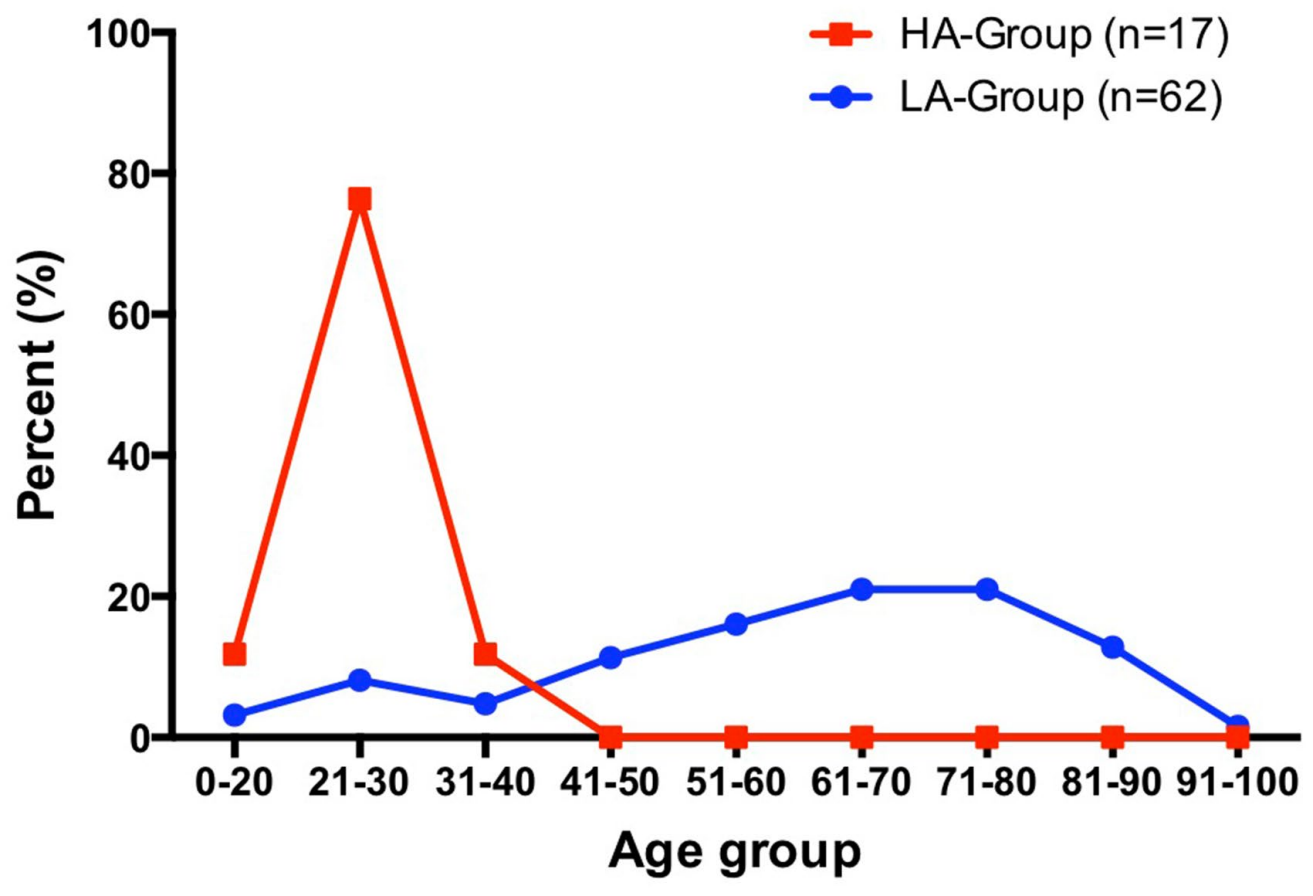
Percentage distribution of patients in HA-Group (total number = 17) and LA-Group (total number = 62) by age interval.

Advanced age is a clear risk factor for VTE, and the risk increases with age, especially after 40 years (4). As age increases, abnormalities in coagulation function and the induction of underlying disease may lead to an increased incidence of VTE. In this study, the distribution of different age groups among LA-Group patients followed this pattern (Figure 4). In contrast, the HA-Group patients were all young individuals, with the oldest being 31 years old, and they had significantly fewer underlying disease than did the LA-Group. This could be due to a difference in the age distribution between the population residing in high-altitude areas and the general population, with a significant reduction in the proportion of older adult individuals, resulting in a predominantly young population affected by the disease. Although this study cannot determine the exact age distribution of the baseline population in the HA-Group, investigations conducted by the research team have revealed that there is also a certain number of older adult individuals, albeit in a relatively smaller proportion. These findings suggest that the low-risk group of young individuals, who normally have a lower incidence of PE, may experience a significantly higher incidence of PE after relocating to high-altitude regions, which is distinctly different from what occurs in the plain areas. As for the specific reasons causing this manifestation, further exploration is still needed.

There were no significant differences in clinical symptoms between the two groups, and only the frequency of occurrence of individual symptoms was different. However, in high-altitude areas, the diagnosis of PE is easily confused with that of altitude-related diseases, such as HAPE (11, 16, 17). Misdiagnosis caused by nonspecific symptoms can delay diagnosis, and the treatment of acute high altitude diseases such as dehydration and diuresis may further promote or worsen venous thrombus formation due to nonspecific symptoms.

In this study, a comparison of laboratory examination data between the two groups revealed that the HA-Group had significantly greater prothrombin time (PT), thrombin time (TT), and activated partial thromboplastin time (APTT) than did the LA-Group. Previous studies have suggested that exposure to high altitudes can lead to hypocoagulable and fibrinolytic system disorders (18–20). However, due to the influence of factors such as different altitudes, the length of time of entering and staying in the plateau, the study population, and exercise load, different studies yield somewhat inconsistent results (21). Currently, there is no unified consensus regarding the impact of hypoxia on the coagulation and fibrinolysis systems. Based on the data of this study, We propose a possible mechanism hypothesis that factors such as the damage to vascular endothelium caused by continuous hypoxic stimulation activate the coagulation and fibrinolysis systems. Nevertheless, the degree of such activation is insufficient to trigger thrombus formation. It is precisely the continuous and low-level activation of both the positive and negative regulatory systems during this ongoing thrombus-formation process that leads to the consumption of coagulation factors and insufficient synthesis in the body (in the plateau area, there are issues like a single-food structure and a decline in gastrointestinal function). Consequently, APTT and PT show an increasing trend. Generally speaking, the body’s stress regulation in response to exposure to a plateau environment is complex and variable. In particular, field studies on human populations are affected by numerous factors.

Notably, the uric acid levels in the HA-Group were significantly higher than those in the LA-Group. Research has confirmed that high-altitude exposure can lead to abnormalities in uric acid production and excretion, resulting in increased serum uric acid levels (22, 23). A study focusing on the East Asian region suggested that elevated serum uric acid is a risk factor for venous thrombosis (24), and other related research has also indicated a positive correlation between increased serum uric acid levels and the risk of venous thrombotic diseases (25–27). Elevated serum uric acid levels can lead to enhanced oxidative stress, causing an increase in reactive oxygen species (ROS), which in turn results in damage to and dysfunction of the vascular endothelium, and further activates platelets and the coagulation cascade (28, 29). Uric acid crystals can also activate the NLRP3 inflammasome, leading to the release of pro-inflammatory cytokines such as interleukin-1β (IL-1β) and interleukin-6 (IL-6), activating the expression of tissue factor and promoting thrombus formation (28).

In this study, the HA-Group consisted of young individuals who moved from plains to high altitudes, which transformed a low-risk population for venous thrombotic diseases into a susceptible group. Could the underlying mechanism be that hypoxia exposure leads to an increase in serum uric acid levels, thereby increasing the risk of venous thrombosis? This potential mechanism is worth further exploration.

The time from the onset of symptoms to a definite diagnosis in the HA-Group was significantly greater than that in the LA-Group. Factors such as inadequate allocation of medical resources, lack of disease recognition ability among medical personnel, and transportation inconvenience may contribute to these results. Both groups of patients primarily received oral anticoagulant therapy (including warfarin, dabigatran and rivaroxaban) outside the hospital. Survival curve analysis revealed a significant difference in the incidence of complete pulmonary artery thrombosis between the HA-Group and LA-Group, suggesting a better overall prognosis for patients in the HA-Group. This may be because the coagulation dysfunction caused by hypoxia is quickly corrected after the patient leaves the high-altitude environment, without further maintenance or exacerbation of thrombus formation. This assumption, of course, is based on the premise that there is no underlying disease that can cause coagulation dysfunction. In contrast, older adult patients have some underlying conditions that lead to a hypercoagulable state (such as tumors, long-term immobilization, advanced age, infections, etc.), and after anticoagulant therapy, they are still unable to rapidly restore normal coagulation mechanisms. Therefore, compared with young people, the older adult population has a relatively delayed resorption rate of blood clots. The above conclusions are based only on this phenomenon, and further research is needed to clarify the specific underlying mechanism involved.

This study has several limitations. First, this was a retrospective single-center observational study in which the medical records of existing patients were evaluated. Therefore, after filtering the case data, the sample size was relatively small, especially in the HA-Group. This is due to the scarcity of people who have migrated to extremely high altitude areas (>5,000 m), which made it valuable to obtain such a sample size. Second, there is a lack of testing for genetic or endogenous factors (such as plasminogen deficiency, protein S/C and variations in related genes) related to PE in patients’ hematological samples. If relevant data could be obtained, they could provide a better evaluation of the risk of developing PE in these two regions.

## Conclusion

In this retrospective study, we included patients with PE from high-altitude and low-altitude areas and analyzed demographic, clinical laboratory, and prognostic data. The results suggest that high-altitude exposure increases the susceptibility of young individuals to PE, while anticoagulant therapy leads to a better prognosis. Abnormal serum uric acid metabolism may be a potential triggering factor for the increased incidence of PE in high-altitude areas, but additional research is needed.

## Data Availability

The original contributions presented in the study are included in the article/supplementary material, further inquiries can be directed to the corresponding authors.
